# Functional and Structural Impairments in the Perirhinal Cortex of a Mouse Model of CDKL5 Deficiency Disorder Are Rescued by a TrkB Agonist

**DOI:** 10.3389/fncel.2019.00169

**Published:** 2019-04-30

**Authors:** Elisa Ren, Vincenzo Roncacé, Stefania Trazzi, Claudia Fuchs, Giorgio Medici, Laura Gennaccaro, Manuela Loi, Giuseppe Galvani, Keqiang Ye, Roberto Rimondini, Giorgio Aicardi, Elisabetta Ciani

**Affiliations:** ^1^Department of Biomedical and Neuromotor Sciences, University of Bologna, Bologna, Italy; ^2^Department for Life Quality Studies, University of Bologna, Bologna, Italy; ^3^School of Medicine, Emory University, Atlanta, GA, United States; ^4^Department of Biomedical and Clinical Sciences, University of Bologna, Bologna, Italy; ^5^Interdepartmental Center “Luigi Galvani” for Integrated Studies of Bioinformatics, Biophysics and Biocomplexity, University of Bologna, Bologna, Italy

**Keywords:** CDKL5, synaptic plasticity, TrkB, PLCγ1, dendritic pattern, GluA2, perirhinal cortex, rett syndrome

## Abstract

Cyclin-dependent kinase-like 5 (CDKL5) deficiency disorder (CDD) is a severe X-linked neurodevelopmental encephalopathy caused by mutations in the *CDKL5* gene and characterized by early-onset epilepsy and intellectual and motor impairments. No cure is currently available for CDD patients, as limited knowledge of the pathology has hindered the development of therapeutics. *Cdkl5* knockout (KO) mouse models, recently created to investigate the role of CDKL5 in the etiology of CDD, recapitulate various features of the disorder. Previous studies have shown alterations in synaptic plasticity and dendritic pattern in the cerebral cortex and in the hippocampus, but the knowledge of the molecular substrates underlying these alterations is still limited. Here, we have examined for the first time synaptic function and plasticity, dendritic morphology, and signal transduction pathways in the perirhinal cortex (PRC) of this mouse model. Being interconnected with a wide range of cortical and subcortical structures and involved in various cognitive processes, PRC provides a very interesting framework for examining how CDKL5 mutation leads to deficits at the synapse, circuit, and behavioral level. We found that long-term potentiation (LTP) was impaired, and that the TrkB/PLCγ1 pathway could be mechanistically involved in this alteration. PRC neurons in mutant mice showed a reduction in dendritic length, dendritic branches, PSD-95-positive puncta, GluA2-AMPA receptor levels, and spine density and maturation. These functional and structural deficits were associated with impairment in visual recognition memory. Interestingly, an *in vivo* treatment with a TrkB agonist (the 7,8-DHF prodrug R13) to trigger the TrkB/PLCγ1 pathway rescued defective LTP, dendritic pattern, PSD-95 and GluA2-AMPA receptor levels, and restored visual recognition memory in *Cdkl5* KO mice. Present findings demonstrate a critical role of TrkB signaling in the synaptic development alterations due to CDKL5 mutation, and suggest the possibility of TrkB-targeted pharmacological interventions.

## Introduction

Cyclin-dependent kinase-like 5 (CDKL5) deficiency disorder (CDD) is a rare encephalopathy characterized by early-onset intractable epileptic seizures, severe intellectual disability, gross motor impairment, stereotypies, visual impairments and autistic-like features (Kalscheuer et al., 2003; Weaving et al., 2004; Bahi-Buisson et al., 2008; Bahi-Buisson and Bienvenu, 2012; Moseley et al., 2012; Paine et al., 2012; Stalpers et al., 2012; Fehr et al., 2013). CDD is caused by mutations in the X-linked gene, *CDKL5*, a member of a highly conserved family of serine-threonine kinases (Tao et al., 2004). To date, several different mutations have been described in the *CDKL5* gene, mainly located within the CDKL5 catalytic domain (Kilstrup-Nielsen et al., 2012; Das et al., 2013; Fehr et al., 2013), suggesting that impaired CDKL5 kinase activity plays an important role in the pathogenesis of CDD (Tao et al., 2004; Bahi-Buisson et al., 2012).

No cure is currently available for CDD patients, as limited knowledge of the pathology has hindered the development of therapeutics. *Cdkl5* knockout (KO) mice (Wang et al., 2012; Amendola et al., 2014; Okuda et al., 2017) have recently been created to investigate the role of CDKL5 in the etiology of CDD. *Cdkl5* KO mice recapitulate different features of CDD, exhibiting severe impairment in learning and memory, visual and respiratory deficits, and motor stereotypies (Wang et al., 2012; Amendola et al., 2014; Fuchs et al., 2014, 2015; Mazziotti et al., 2017). The neuropathology of CDD points to arrested neuronal development rather than neurodegeneration or severe malformation of nervous tissue. Reduced neuronal branching and spine density have been observed in the visual and somatosensory cortex (Della Sala et al., 2016; Pizzo et al., 2016), and in the hippocampal region (Amendola et al., 2014; Fuchs et al., 2014) of *Cdkl5* KO mice. In addition, immunocytochemical studies have demonstrated alterations in synaptic connectivity, which might lead to an excitation-inhibition imbalance (Pizzo et al., 2016; Sivilia et al., 2016). Together, these observations have led to the suggestion that there is an overall reduction in the number of synaptic inputs to neurons in the CDKL5-deficient brain. It has been shown that CDKL5 is localized at excitatory synapses (Ricciardi et al., 2012) where it binds to the scaffolding postsynaptic density protein 95 (PSD-95) and to the synaptic cell adhesion molecule NGL-1 (Ricciardi et al., 2012; Zhu et al., 2013). Cdkl5 deficiency in primary hippocampal neurons leads to deranged expression of the GluA2 subunit of alpha-amino-3-hydroxy-5-methyl-4-isoxazole propionic acid receptors (GluA2-AMPAR), and it is probable that this prompts an alteration of synaptic functions (Tramarin et al., 2018).

To date, only a few studies have investigated the functional consequences of these changes at the synaptic level (Della Sala et al., 2016; Okuda et al., 2017; Tang et al., 2017). Long-term potentiation (LTP) is the most widely studied form of synaptic plasticity in the mammalian nervous system. It provides a neuronal substrate for learning and memory and is impaired in several models of psychiatric and neurologic disorders. LTP was found to be altered in opposing ways in two brain regions of *Cdkl5* KO mice: it was strongly reduced in the somatosensory cortex (Della Sala et al., 2016) and slightly increased in the hippocampal CA1 region (Okuda et al., 2017). The latter effect appears to be mediated by upregulation of GluN2B-containing NMDA receptors (Okuda et al., 2017). Interestingly, the ablation of Cdkl5 expression specifically from forebrain glutamatergic neurons leads to impairment in hippocampal neuronal maturation, synaptic function, and impairment in hippocampal-dependent learning and memory (Tang et al., 2017). Despite this well-documented evidence, knowledge of the molecular substrates underlying CDKL5-related alterations in synaptic plasticity is still limited.

The perirhinal cortex (PRC) is located at the boundary between the medial temporal lobe and the ventral visual pathway. It has several interconnections with a wide range of cortical and subcortical structures and is involved in various cognitive processes. In particular, it plays an essential role in visual recognition memory, that is critical to the ability to record events and to guide prospective behavior (Kealy and Commins, 2011; Suzuki and Naya, 2014; Brown and Banks, 2015). A previous study has shown that male *Cdkl5* KO mice tested in a sociability protocol are more interested than littermate controls in a novel object (Wang et al., 2012). This might suggest that visual recognition memory is not affected by CDKL5 mutation, but a novel object recognition (NOR) test is required to exclude this possibility.

Perirhinal cortex has never been investigated in *Cdkl5* KO mice. Here we provide evidence for LTP impairment, associated with reduced dendritic length, dendritic branches, PSD-95-positive puncta, GluA2-AMPA receptor levels, and spine density and maturation. Data obtained in a four-object NOR indicate that also visual recognition memory is impaired. Notably, most of these alterations, including LTP and visual recognition memory impairments, were rescued by triggering the TrKB/PLCγ1 pathway using the 7,8-DHF prodrug R13.

## Materials and Methods

### Colony

The mice were produced by crossing *Cdkl5* KO +/- females with *Cdkl5* KO Y/- males (Amendola et al., 2014). Littermate controls were used for all experiments. Animals were karyotyped using PCR on genomic DNA as previously described (Amendola et al., 2014). The day of birth was designated as postnatal day (P) zero and animals with 24 h of age were considered as 1-day-old animals (P1). Mice were housed three to five per cage on a 12-h light/dark cycle in a temperature-controlled environment with food and water provided *ad libitum*. Experiments were performed in accordance with the European Communities Council Directive of 24 November 1986 (86/609/EEC) for the use of experimental animals, and were approved by the Italian Ministry of Public Health (approval n 114/2018-PR). All efforts were made to minimize animal suffering and to keep the number of animals used to a minimum.

### Experimental Protocol

Experiments were carried out on a total of 85 *Cdkl5* -/Y and 70 *Cdkl5* +/Y mice. Treated *Cdkl5* -/Y and *Cdkl5* +/Y mice received a daily intraperitoneal injection (at 9–10 am) of R13 (7,8-DHF prodrug (Chen et al., 2018), 5.0 mg/kg in vehicle: PBS with 1% DMSO) or vehicle from P35 to P50. The dosage of 5.0 mg/kg R13 (7,8-DHF) was chosen on the basis of previous *in vivo* studies (Jang et al., 2010; Liu et al., 2010; Andero et al., 2011, 2012; Devi and Ohno, 2012), which demonstrated central TrkB activation enhanced neurogenesis and related behavioral changes in rodents treated with systemic 7,8-DHF administration. Intraperitoneal administration was chosen since prolonged oral gavage administration was stressful and harmful for *Cdkl5* KO mice. At the end of the treatment, (P50) mice were sacrificed for electrophysiological or histological analyses or behavioral testing.

### Electrophysiology

Preparation of horizontal brain slices (400 μm-thick, including the PRC, the entorhinal cortex and the hippocampus), electrophysiological recording of evoked field excitatory postsynaptic potentials (fEPSP), measurements of fEPSP amplitude and calculation of paired-pulse ratio (PPR) were performed as previously reported (Ziakopoulos et al., 1999; Aicardi et al., 2004; Roncacé et al., 2017) and are described in detail in the online Supplementary Data. Theta burst stimulation (TBS; four trains every 15 s, each train comprising 10 bursts of 5 pulses at 100 Hz, inter-burst interval 150 ms) (Ziakopoulos et al., 1999) was used to induce LTP. Synaptic plasticity was further investigated by delivering four consecutive TBS stimulations at 15-min intervals (Weng et al., 2011).

### Immunohistochemistry

Some animals were deeply anesthetized and transcardially perfused with ice cold phosphate-buffered saline (PBS, 100 mM, pH 7.4), followed by a 4% solution of paraformaldehyde in PBS. The right hemisphere was cut with a freezing microtome into 30 μm-thick coronal sections.

#### Synaptic Terminals

One out of four sections (3–6 per mouse) of the PRC was used for immunohistochemistry. Free-floating sections were stained overnight at 4°C with the primary antibodies post-synaptic density protein 95 (1:1000, anti-PSD-95 rabbit polyclonal Ab, Abcam, Cambridge, United Kingdom) or glutamate vesicular transporter 1 (1:500, anti-VGlut1 rabbit polyclonal Ab, Thermo Scientific), and then stained in fluorescent secondary antibody (Cy3-conjugated anti-rabbit secondary antibody 1:200; Jackson ImmunoResearch Laboratories Inc., West Grove, PA, United States) for 2 h at room temperature. For quantification of synaptic puncta, images immunoprocessed for PSD-95 and VGlut1 were acquired with a Leica TCS SL confocal microscope. In each section four images from the PRC were captured and the density of individual puncta exhibiting VGlut1 or PSD-95 immunoreactivity was evaluated as previously described (Guidi et al., 2013).

#### P-PLCγ1 Intensity

Horizontal brain slices were cut from both hemispheres, and LTP was induced in the PRC by TBS as described above and in online Supplementary Data (Electrophysiology). Ten minutes after TBS, the slices were fixed in a 4% solution of paraformaldehyde in PBS, then cryoprotected in 15–20% sucrose. Brain slices were cut using a freezing microtome into 30 μm-thick transversal sections. Free-floating sections were stained overnight at 4°C with the primary antibodies P-PLCγ1 (1:500, anti-P-PLCγ (Y783) rabbit polyclonal Ab, Cell Signaling Technology, Danvers, MA, United States), and then stained in fluorescent secondary antibody (Cy3-conjugated anti-rabbit secondary antibody 1:200; Jackson ImmunoResearch Laboratories, Inc., West Grove, PA, United States) for 2 h at room temperature. Nuclei were counterstained with Hoechst-33342 (Sigma-Aldrich) and fluorescence images were acquired at the same intensity. To assess P-PLCγ1 cytoplasmic intensity a cytoplasmic area was traced and the Cy3-staining corresponding to the P-PLCγ1 signal was quantified by determining the mean intensity of positive (bright) pixels inside this area. The intensity of the cytoplasmic signal was normalized to the background outside the same cell by calculating the ratio between the intensity of cytoplasmic versus extra cellular signal.

### Golgi Staining

Golgi staining, measurement of the dendritic tree, and dendritic spine analysis and calculation were performed as previously reported (Guidi et al., 2013; Risher et al., 2014), and are described in detail in the online Supplementary Data.

### Western Blotting

In order to obtain samples of the PRC in isolation, it was micro-dissected from slices (400 μm-thick) taken at the same levels as those used for electrophysiological recording. In homogenates of the PRC of P50 mice, total proteins were obtained as previously described (Trazzi et al., 2011) and the antibodies used are listed in Supplementary Table. For TrkB, PLCγ1, and Erk phosphorylation levels, membranes were probed with the antibody for the phosphorylated form of the analyzed protein, stripped with the Restore^TM^ Stripping Buffer (Thermo Fisher Scientific) following manufacturer’s instructions, and then re-probed with the antibody for the un-phosphorylated form of the same protein. Densitometric analysis of digitized images was carried out with ChemiDoc XRS Imaging Systems and Image LabTM Software (Bio-Rad Laboratories, Hercules, CA, United States).

### Behavioral Testing

The animal behavioral test was performed by operators blind to genotype and treatment. Mice were allowed to habituate to the testing room for at least 1 h before the test, and testing was always performed at the same time of day. The test was performed in an open field-arena (50 × 50 cm) and the behavior of the mice was monitored using a video camera placed above the center of the arena. The experiments were scored using EthoVision XT ver. 14 software (Noldus, Netherlands). Test chambers were cleaned with 70% ethanol between test subjects.

#### Pretraining Habituation

The animals were habituated in the open field arena without stimuli for 2 days before the commencement of the behavioral testing. Each animal was placed in the center of the arena and allowed to freely explore the open field for 20 min.

#### Novel Object Preference Task

The procedure involved a familiarization phase, followed by a preference test phase (Supplementary Figure 1A). In the familiarization phase (10 min duration), each animal was placed in the same arena (of the pre-training habituation), in which four copies of the same object (a plastic tube, too heavy for the animal to displace; objects 1–4) were located near the four corners of the arena (15 cm from each adjacent wall). After 1 h delay, during which one of the four objects (object 1) was replaced by a novel object (a wooden cube; objects 2–4 remained in the same positions), the animal was returned to the arena for the preference test phase (10 min duration; Supplementary Figure 1A).

#### Behavioral Measures

Exploration behavior was defined as the animal directing its nose toward the object at a distance ≤ 2 cm or touching it with the nose, while turning around or sitting on the object was not considered as an exploration. Discrimination among the objects was calculated using the Exploratory Preference Index, i.e., the percentage of time spent exploring any of the four objects over the total time spent exploring the four objects (Wang et al., 2007). Therefore, a preference index of above 25% indicates the preference for an object. Data obtained from the 10 min of the test period are presented.

### Statistical Analysis

Results are presented as the mean ± standard error of the mean (SE). Statistical testing was performed using the two-tailed Student’s *t*-test or two-way ANOVA with genotype (*Cdkl5* -/Y, *Cdkl5* +/Y) and treatment (R13, vehicle) as factors, followed by Fisher’s LSD or the Tukey *post hoc* test. For categorical data, that is, percentages of spines, we used a chi-squared test. A probability level of *p* < 0.05 was considered to be statistically significant.

## Results

### LTP Is Impaired in the Perirhinal Cortex of *Cdkl5* KO Mice

**FIGURE 1:**
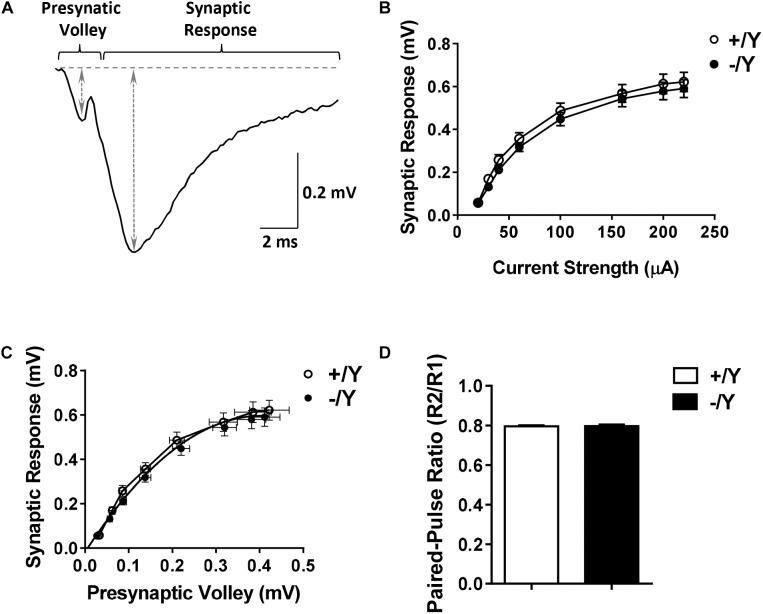
Input-output relations and responses to paired-pulse stimulation in the perirhinal cortex (PRC) of *Cdkl5* +/Y and *Cdkl5* -/Y mice. **(A)** Example of the response evoked in layers II/III of the PRC by stimulation of layers II/III. The double-headed arrows indicate how the magnitude of the presynaptic volley and synaptic response were measured. **(B)** Magnitude of the synaptic response as a function of the stimulus strength in P50 *Cdkl5* +/Y (*n* = 16; 10 animals) and *Cdkl*5 -/Y (*n* = 21; 10 animals) mice. **(C)** Magnitude of the synaptic response as a function of the magnitude of the presynaptic volley. **(D)** Ratio of the synaptic responses (R2/R1) evoked by a pair of stimuli with an interpulse interval of 200 ms in P50 *Cdkl5* +/Y (*n* = 11; 9 animals) and *Cdkl5* -/Y (*n* = 9; 9 animals) mice. Values represent mean ± SE.

To evaluate possible differences in PRC functional connectivity between *Cdkl5* -/Y and wild-type (+/Y) mice, we compared fEPSPs evoked in layers II–III of the PRC by stimuli applied in the same layers (Figure 1A). Stimulus-response curves (input-output relationships) obtained from *Cdkl5* -/Y slices were not significantly different from those of wild-type mice over a wide range of stimulus intensities (Figure 1B). Thus, the basic properties of synaptic function in response to single stimuli appear to be unaltered by *Cdkl5* deletion. This conclusion is strengthened by the observation that the slices from *Cdkl5* -/Y and wild-type (+/Y) mice exhibited a similar relationship between the magnitude of the afferent volley (“non-synaptic” component of the response) and the magnitude of the synaptic response (Figure 1C). Also the responses to paired stimuli obtained in slices from *Cdkl5* -/Y mice were not significantly different from those of wild-type mice (Figure 1D), suggesting that *Cdkl5* deletion does not affect short-term synaptic plasticity.

**FIGURE 2:**
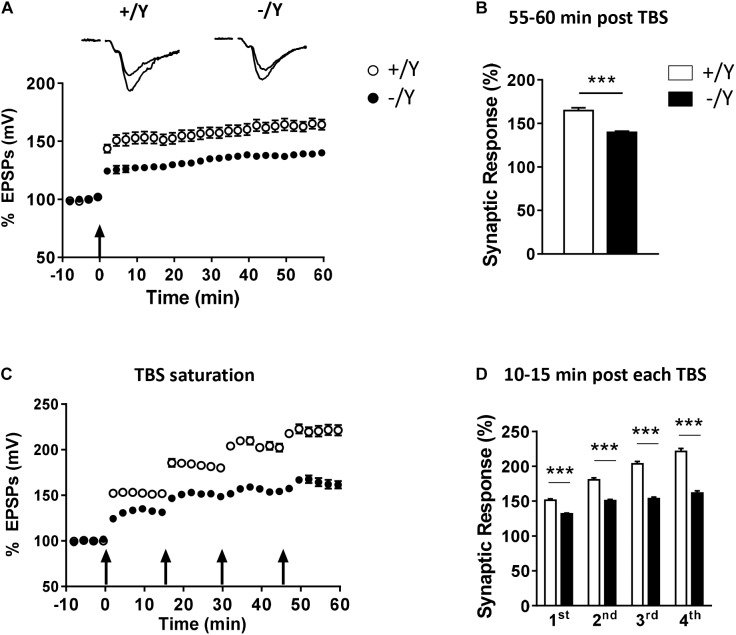
LTP in the perirhinal cortex of *Cdkl5* +/Y and *Cdkl5* -/Y mice. **(A)** Amplitude of the synaptic responses evoked before and after theta burst stimulation (TBS; four trains every 15 s, each train comprising 10 bursts of 5 pulses at 100 Hz, inter-burst interval 150 ms). Here and in the following panels **(B–D)**, the amplitude of the responses is expressed as a percentage of the average amplitude of responses recorded 10 min before LTP induction. Here, and in panel C, the arrows indicate the time of delivery of the TBS. The traces at the top are examples of responses recorded before, and 55–60 min after, LTP induction. **(B)** The histograms indicate the averaged amplitude of the responses recorded 55–60 min after TBS. Same data as in **(A)**. Recordings were carried out in slices from P50 *Cdkl5* +/Y (*n* = 9; 5 animals) and *Cdkl5* -/Y (*n* = 11; 9 animals) mice. **(C)** Amplitude of the synaptic response evoked before and after four consecutive TBS stimulations delivered at 15-min intervals. **(D)** The histograms indicate the averaged amplitude of the responses recorded 10–15 min after each TBS. Same data as in **(C)**. Recordings were carried out in slices from P50 *Cdkl5* +/Y (*n* = 7; 7 animals) and *Cdkl5* -/Y (*n* = 7; 7 animals) mice. ^∗∗∗^*p* < 0.001 (Student’s two-tailed *t*-test).

To evaluate the possibility that *Cdkl5* deletion may affect long-term synaptic plasticity, we induced LTP in layers II–III of the PRC using TBS, i.e., short trains of 100 Hz pulses delivered at theta (5 Hz) frequency. We found that the magnitude of TBS-induced LTP was significantly smaller in slices from *Cdkl5* -/Y mice (Figure 2A,B). Figure 2B shows that 55–60 min after TBS the LTP magnitude was 165 ± 3.1% in slices from wild-type (+/Y) mice, and only 139.0 ± 1.6% in slices from *Cdkl5* -/Y mice. Further investigation of synaptic plasticity was conducted by delivering four consecutive TBS stimulations at 15-min intervals, in order to reveal possible saturation effects. Figure 2C,D show that the four TBS stimulations induced a significantly smaller progressive increase of LTP magnitude in slices from *Cdkl5* -/Y mice compared to slices from wild-type (+/Y) mice, revealing a saturation effect after the second stimulation (+/Y: 151.3 ± 2.1%, 180.8 ± 2.5%, 203.7 ± 3.3% and 221.4 ± 4.2%; -/Y: 131.7 ± 1.2%, 150.7 ± 1.8%, 153.6 ± 2.3% and 161.5 ± 3.3% of baseline response; Figure 2D).

### TrkB/PLCγ1 Signaling Is Impaired in the Perirhinal Cortex of *Cdkl5* KO Mice

**FIGURE 3:**
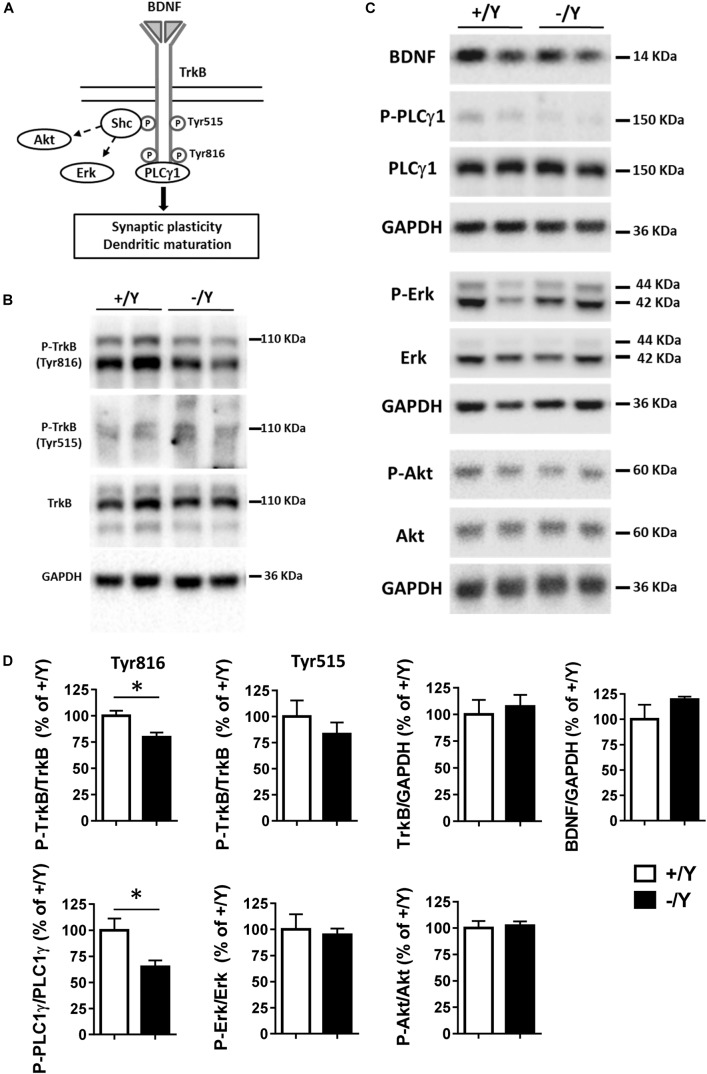
TrkB signaling pathways in the perirhinal cortex of *Cdkl5* +/Y and *Cdkl5* -/Y mice. **(A)** Diagram of brain-derived neurotrophic factor (BDNF) and TrkB signaling pathways. BDNF binds to the extra cellular domain of TrkB forming homodimers to activate downstream intracellular signaling cascades, including Shc/Erk, Shc/Akt, and phospholipase C (PLC) γ1 pathways. **(B)** Western blots examples of P-TrkB (Tyr816 and Tyr515) and TrkB in PRC homogenates of P50 *Cdkl5* +/Y and *Cdkl5* -/Y mice. **(C)** Western blot examples of BDNF, P-PLCγ1 (Tyr783), PLCγ1, P-Erk, Erk, P-Akt (Ser437), Akt and GAPDH levels in PRC homogenates of P50 *Cdkl5* +/Y and *Cdkl5* -/Y mice. **(D)** The histograms show western blot analysis of samples as in B,C. Phosphoprotein levels were normalized to corresponding total protein levels, and total protein levels were normalized to GAPDH (*Cdkl5* +/Y *n* = 5; *Cdkl5* +/Y *n* = 5). Data are expressed as % of those obtained in *Cdkl5* +/Y mice. Values represent mean ± SE. ^∗^*p* < 0.05 (Student’s two-tailed *t*-test).

A previous study in the PRC has shown that TBS elicits a large increase in brain-derived neurotrophic factor (BDNF) secretion, which is necessary for LTP induction (Aicardi et al., 2004). As shown in Figure 3A, BDNF acts on TrkB receptors leading to dimerization and autophosphorylation of tyrosine residues at position Tyr515 and Tyr816. Phosphorylation and recruitment of adaptors at position 515 activate Erk and Akt pathways, which promote survival and growth of neurons and other cells through Ras or GRB-associated binder G1 (GAB1). Phosphorylation at position 816 recruits and activates phospholipase Cγ1 (PLCγ1), which results in the generation of inositol-1,4,5-triphosphate and diacylglycerol, leading to synaptic plasticity and dendritic maturation (Minichiello et al., 2002; Gartner et al., 2006; Minichiello, 2009).

To investigate the molecular mechanisms underlying TBS-induced LTP impairment, we first analyzed the levels of TrkB phosphorylation using Western blot in tissue homogenates isolated from the PRC. *Cdkl5* -/Y mice showed no differences in the P-TrkB(Tyr515) or total TrkB protein levels (Figure 3B,D). On the contrary, we found a significantly lower level of P-TrkB(Tyr816) protein in *Cdkl5* -/Y mice compared to wild-type (+/Y) mice (Figure 3B,D). No significant differences in BDNF protein level were observed (Figure 3C,D), suggesting a specific effect of *Cdkl5* deletion on the autophosphorylation process at position Tyr816 of TrkB, rather than on BDNF availability. We then examined the main downstream effectors of the TrkB pathway. Predictably, we found a significantly lower PLCγ1 phosphorylation in the PRC of *Cdkl5* KO mice in comparison with wild-type mice, whereas no significant differences in Erk and Akt phosphorylation were observed (Figure 3C,D).

### Impaired PLCγ1 Phosphorylation and LTP in the Perirhinal Cortex of *Cdkl5* KO Mice Are Rescued by Treatment With the TrkB Agonist R13

**FIGURE 4:**
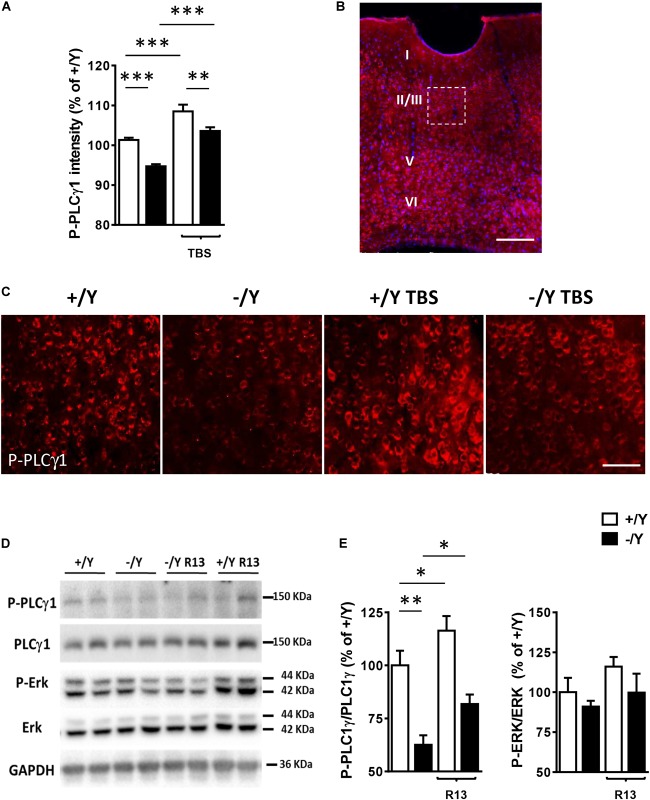
Effect of TBS or treatment with R13 on TrkB/ PLCγ1 signaling in the perirhinal cortex of *Cdkl5* -/Y mice. **(A)** Quantification of P-PLCγ1 signal intensity in PRC slices before and after theta burst stimulation (TBS; four trains every 15 s, each train comprising 10 bursts of 5 pulses at 100 Hz, inter-burst interval 150 ms) of *Cdkl5* +/Y (*n* = 4) and *Cdkl5* -/Y (*n* = 4) mice. **(B)** A representative image of PRC processed for fluorescent P-PLCγ1 immunostaining (red) of a wild-type (+/Y) mouse. Nuclei are stained with Hoechst (blue). The dotted box indicates the region shown at a higher magnification in **(C)**. Scale bar = 50 μm. Roman numerals indicate PRC cytoarchitectonic layers. **(C)** Representative images of layer II-III PRC neurons of *Cdkl5* +/Y and *Cdkl5* -/Y PRC slices as in **(A)**. **(D,E)** Quantification of PLCγ1 and Erk phosphorylation levels before and after treatment with R13. *Cdkl5* -/Y and *Cdkl5* +/Y mice were treated for 15 days (5 mg/Kg IP) from postnatal day 35 (P35) to 50 (P50), the day of sacrifice. Western blot examples **(D)** and analyses **(E)** of P-PLCγ1 (Tyr783), PLCγ1, P-Erk and Erk levels in PRC homogenates of vehicle-treated *Cdkl5* +/Y (*n* = 5) and *Cdkl5* -/Y (*n* = 5) mice, and R13-treated *Cdkl5* +/Y (*n* = 6) and *Cdkl5* -/Y (*n* = 6) mice. Values represent mean ± SE. ^∗^*p* < 0.05; ^∗∗^*p* < 0.01; ^∗∗∗^*p* < 0.001 (Fisher’s LSD test after two-way ANOVA).

In order to investigate whether TrkB/PLCγ1 signaling alteration underlies LTP impairment in *Cdkl5* KO mice, we quantified P-PLCγ1 cellular intensity in PRC slices from *Cdkl5* KO and wild-type mice before and after TBS. Figure 4A–C show that 10 min after TBS, PLCγ1 phosphorylation was increased in PRC slices from both *Cdkl5* and wild-type mice. Interestingly, PLCγ1 phosphorylation after TBS was still significantly lower in *Cdkl5* KO slices than in wild-type slices.

In order to investigate the effect of chronic activation of TrkB/PLCγ1 signaling in *Cdkl5* KO mice, we treated *Cdkl5* -/Y mice for 15 days with R13, a prodrug of 7,8-dihydroxyflavone (7,8-DHF) (Chen et al., 2018). 7,8-DHF is a potent molecular TrkB agonist that specifically binds to the TrkB receptor extra cellular domain. A recent study in striatal neurons has shown that it acts through selective phosphorylation of the 816 residue of TrkB, leading to activation of the PLCγ1 pathway (Garcia-Diaz Barriga et al., 2017). Accordingly, we found that R13 treatment increased PLCγ1 phosphorylation levels but not P-Erk levels in both *Cdkl5* and wild-type mice (Figure 4D,E), suggesting a selective action of 7,8-DHF on the TrkB(Y816)-PLCγ1 pathway also in the PRC.

**FIGURE 5:**
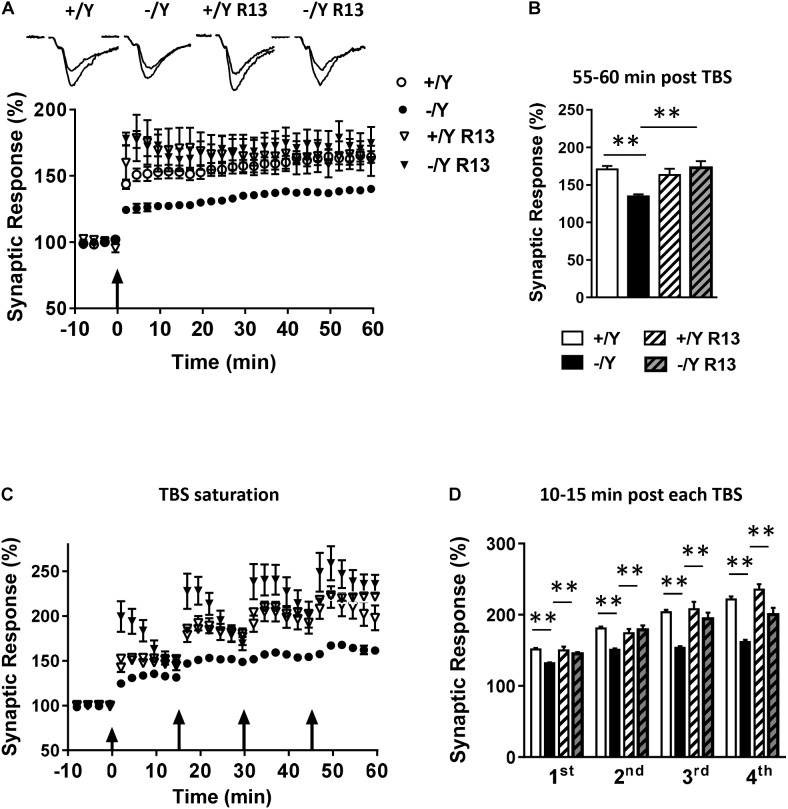
Effect of treatment with R13 on LTP in the perirhinal cortex of *Cdkl5* -/Y mice. **(A)** Amplitude of the synaptic responses evoked before and after TBS. Here, and in the following panels **(B–D)**, the amplitude of the responses is expressed as a percentage of the average amplitude of responses recorded 10 min before TBS. The traces at the top are examples of responses recorded before, and 55–60 min after, TBS. Here, and in panel C, the arrows indicate the time of TBS delivery. Recordings were carried out in slices from P50 vehicle-treated *Cdkl5* +/Y (*n* = 13; 7 animals) and *Cdkl5* -/Y (*n* = 13; 6 animals), and R13-treated *Cdkl5* +/Y (*n* = 6; 4 animals) and *Cdkl5* -/Y (*n* = 7; 7 animals) mice. **(B)** The histograms indicate the averaged amplitude of the responses recorded 55–60 min after TBS. Same data as in **(A)**. **(C)** Amplitude of the synaptic responses evoked before and after four consecutive TBS stimulations delivered at 15-min intervals. Recordings were carried out in slices from P50 vehicle-treated *Cdkl5* +/Y (*n* = 6; 6 animals) and *Cdkl5* -/Y (*n* = 7; 7 animals), and R13-treated *Cdkl5* +/Y (*n* = 7; 4 animals) and *Cdkl5* -/Y (*n* = 6; 6 animals) mice. **(D)** The histograms indicate the averaged amplitude of the responses recorded 10–15 min after each TBS. Same data as in **(C)**. Values represent mean ± SE. ^∗^*p* < 0.05; ^∗∗^*p* < 0.01 (Tukey test after two-way ANOVA).

Interestingly, R13 treatment restored LTP in *Cdkl5* -/Y mice (Figure 5A–D), suggesting a critical role of TrkB/PLCγ1 signaling in the defective synaptic plasticity. R13 treatment affected neither LTP in *Cdkl5* +/Y mice (Figure 5A–D) nor basal synaptic transmission (input-output responses; responses to paired stimuli) in *Cdkl5* -/Y and *Cdkl5* +/Y mice (not shown). Vehicle treatment did not affect any functional parameter considered in *Cdkl5* -/Y and *Cdkl5* +/Y mice (Figure 5A–D).

### Alteration of Dendritic Pattern in the Perirhinal Cortex of *Cdkl5* KO Mice Is Rescued by Treatment With the TrkB Agonist R13

BDNF/TrkB signaling participates in the regulation of dendritic differentiation, and in the formation and maturation of dendritic spines during postnatal development (Chapleau et al., 2009). Abnormalities in dendritic and synaptic differentiation are thought to underlie altered synaptic function and network connectivity, thus contributing to the impaired neuronal function. Previous studies in *Cdkl5* -/Y mice have shown a reduction in dendritic pattern in granule cells and CA1 pyramidal neurons of the hippocampal region (Fuchs et al., 2015; Trazzi et al., 2016).

**FIGURE 6:**
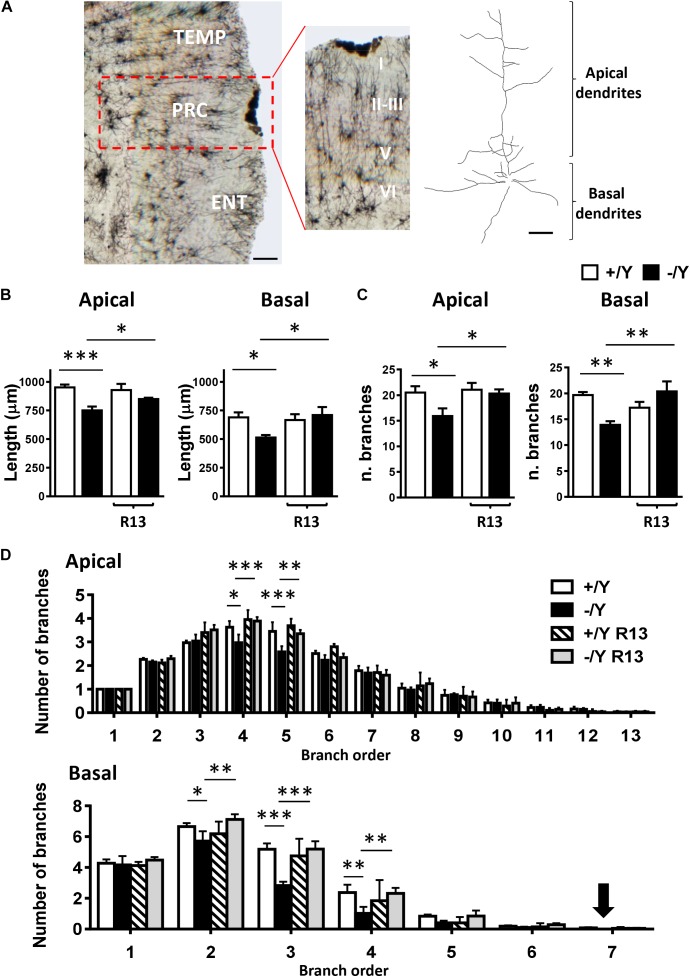
Effect of treatment with R13 on dendritic morphology in the perirhinal cortex of *Cdkl5* -/Y mice. **(A)** Example of Golgi-stained section at the level of the PRC of a P50 *Cdkl5* -/Y mouse (panel on the left; scale bar = 100 μm). The central panel shows a higher magnification of the area enclosed in the red square. Roman numerals indicate PRC cytoarchitectonic layers. The panel on the right shows an example of the apical and basal dendritic tree of a Golgi-stained PRC neuron from layers II-III (scale bar = 30 μm). **(B)** Apical and basal mean total dendritic length of layer II-III PRC neurons of vehicle-treated P50 *Cdkl5* +/Y (*n* = 4) and *Cdkl5* -/Y (*n* = 4) mice, and R13-treated P50 *Cdkl5* +/Y (*n* = 4) and *Cdkl5* -/Y (*n* = 5) mice. **(C)** Mean number of dendritic segments of the apical and basal dendrites of layer II-III PRC neurons in the same mice as in **(B)**. **(D)** Mean number of apical and basal branches of the different orders of vehicle-treated *Cdkl5* +/Y (*n* = 4) and *Cdkl5* -/Y (*n* = 4) mice, and R13-treated *Cdkl5* -/Y (*n* = 5) mice. The numbers on the X axis indicate the branch order. The arrow indicates the absence of branches in *Cdkl5* -/Y mice. Values in **(B–D)** represent mean ± SE. ^∗^*p* < 0.05; ^∗∗^*p* < 0.01; ^∗∗∗^*p* < 0.001 (Fisher’s LSD test after two-way ANOVA).

In order to establish whether these alterations also occur in PRC neurons, we examined apical and basal dendritic branches of layer II-III PRC neurons in Golgi-stained brain sections (Figure 6A). We found that both basal and apical dendrites were shorter (Figure 6B), and there was a reduced number of branches (Figure 6C) in PRC neurons of *Cdkl5* -/Y compared to *Cdkl5* +/Y mice. Interestingly, R13 treatment restored dendritic length and number of branches in *Cdkl5* -/Y mice, but had no effect in control mice (+/Y) (Figure 6B,C). Figure 6D shows that the difference in number of branches was significant in the fourth and fifth order in apical dendrites, and in the second, third, and fourth order in basal dendrites; moreover, it shows a lack of branches of the seventh order in basal dendrites. All these defects were corrected by R13 treatment. These results indicate that activation of the TrkB/PLCγ1 pathway restores the dendritic pattern of the PRC neurons in *Cdkl5* -/Y mice. Vehicle treatment did not affect any morphological parameter considered in *Cdkl5* -/Y and *Cdkl5* +/Y mice (Figure 6B–D).

### Alteration of Dendritic Spine Density in the Perirhinal Cortex of *Cdkl5* KO Mice Is Rescued by Treatment With the TrkB Agonist R13

**FIGURE 7:**
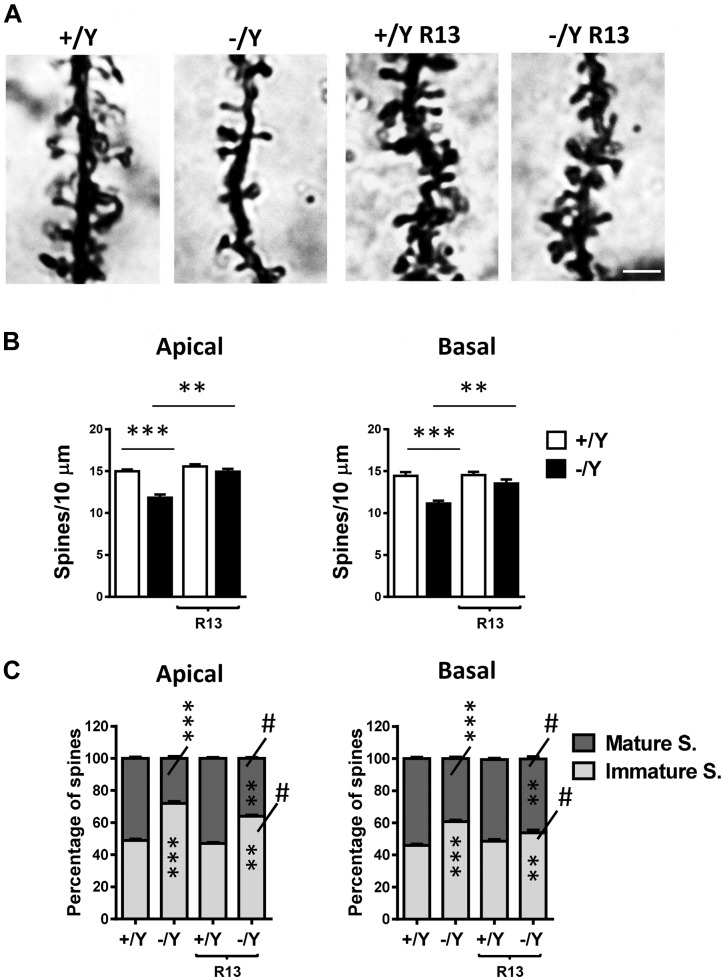
Effect of treatment with R13 on dendritic spines in the perirhinal cortex of *Cdkl5* -/Y mice. **(A)** Images of Golgi-stained dendritic branches of layer II-III perirhinal neurons of a P50 *Cdkl5* +/Y and a *Cdkl5* -/Y mouse, and of a *Cdkl5* -/Y mouse treated with R13. Scale bar = 1.5 μm. **(B)** Dendritic spine density (number of spines per 10 μm) in apical and basal dendrites from vehicle-treated *Cdkl5* +/Y (*n* = 4) and *Cdkl5* -/Y (*n* = 4) mice, and *Cdkl5* +/Y (*n* = 4) and *Cdkl5* -/Y (*n* = 4) mice treated with R13. **(C)** Percentage of immature and mature spines in relation to the total number of protrusions of layer II-III perirhinal neurons in mice as in **(B)**. Values represent mean ± SE. ^∗∗^*P* < 0.01; ^∗∗∗^*P* < 0.001; ^#^*p* < 0.01 compared to untreated *Cdkl5* -/Y mice in **(C)** [data in **(B)** Fisher’s LSD test after two-way ANOVA; data in **(C)** Chi-squared test].

In Golgi-stained brain sections we examined spine density in the apical and basal dendritic branches of layer II-III neurons of the PRC. PRC neurons had a reduced spine density in both basal and apical dendrites in untreated *Cdkl5* -/Y mice in comparison with wild-type (+/Y) mice (Figure 7A,B). *Cdkl5* -/Y mice treated with R13 underwent a restoration of spine density (Figure 7A,B). Dendritic spines are heterogeneous in size and shape, and can be classified as immature spines (filopodia, thin-shaped, and stubby-shaped) and mature spines (mushroom and cup shapes) (Risher et al., 2014). Separate counts of different classes of dendritic spines revealed that PRC neurons of *Cdkl5* -/Y mice had a higher percentage of immature spines and a reduced percentage of mature spines compared to *Cdkl5* +/Y mice (Figure 7C). Treatment with R13 improved the balance between immature and mature spines (Figure 7C), suggesting that activation of the TrkB/PLCγ1 pathway improves PRC dendritic spine maturation. Vehicle treatment did not affect spine density in *Cdkl5* -/Y and *Cdkl5* +/Y mice (Figure 7A,B).

### Altered Expression of PSD-95 PositiveSynaptic Puncta and GluA2-AMPAR inthe Perirhinal Cortex of *Cdkl5* KO Mice IsRescued by Treatment With the TrkBAgonist R13

**FIGURE 8:**
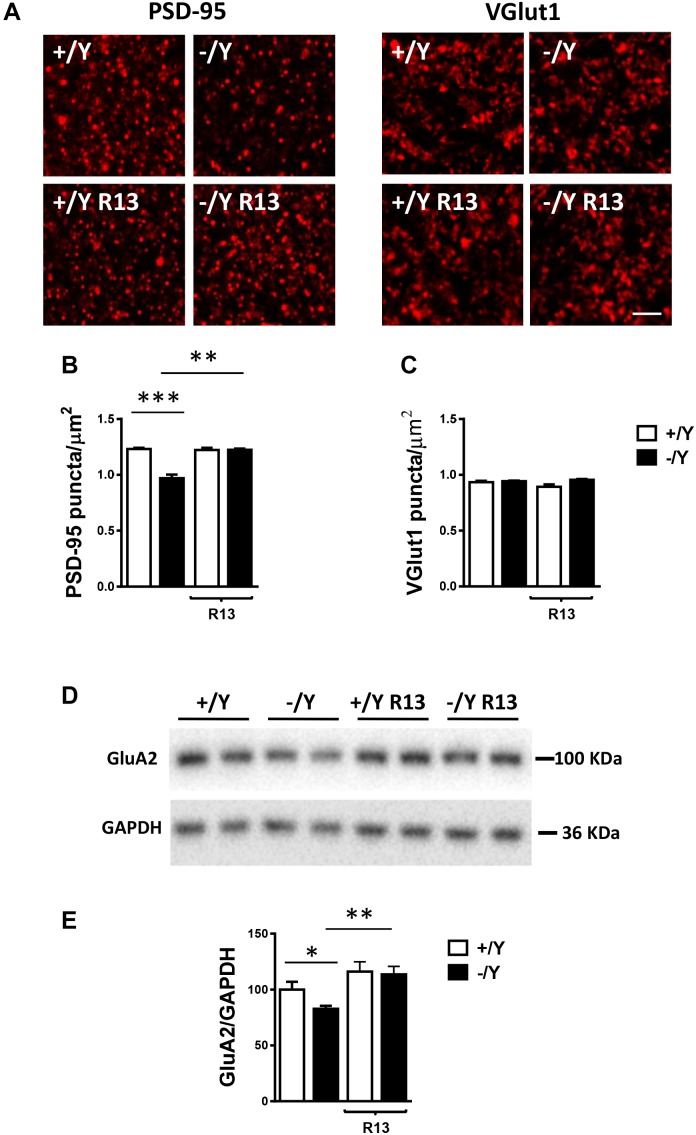
Effect of treatment with R13 on neuronal connectivity in the perirhinal cortex of *Cdkl5* -/Y mice. **(A)** Representative fluorescence image of a PRC section processed for PSD-95 or VGlut1 immunoreactivity, showing examples of PSD-95 and VGlut1 immunoreactive puncta. Scale bar: 3 μm. **(B,C)** Number of fluorescent puncta per μm^2^ exhibiting PSD-95 **(B)** or VGlut1 **(C)** immunoreactivity in layers II-III of the PRC of vehicle-treated P50 *Cdkl5* +/Y (*n* = 4) and *Cdkl5* -/Y (*n* = 4) mice, and P50 *Cdkl5* +/Y (*n* = 4) and *Cdkl5* -/Y (*n* = 4) mice treated with R13. **(D,E)** Western blot analysis of GluA2-AMPA levels in PRC homogenates of vehicle-treated P50 *Cdkl5* +/Y (*n* = 6) and *Cdkl5* -/Y (*n* = 6) mice and R13-treated P50 *Cdkl5* -/Y (*n* = 5) mice. Values represent mean ± SE. ^∗^*p* < 0.05; ^∗∗^*p* < 0.01; ^∗∗∗^*p* < 0.001 (data in **(B,C)** Fisher’s LSD test after two-way ANOVA; data in **(E)** Student’s two-tailed *t*-test).

Most excitatory synapses in the mature mammalian brain occur on spines in which postsynaptic density protein 95 (PSD-95) clusters are localized. Evaluation of PSD-95 immunoreactivity showed a strong reduction in the number of PSD-95-positive puncta in the PRC of *Cdkl5* -/Y mice (Figure 8A,B), which is consistent with the reduced number of mature spines (Figure 7C) and suggests loss of excitatory synaptic contacts. To clarify this issue, we examined the immunoreactivity for the vesicular glutamate transporter-1 (VGlut1), a marker of glutamatergic (excitatory) terminals. We found no difference in the number of VGlut1-positive immunoreactive puncta between *Cdkl5* -/Y and wild-type mice (Figure 8A,C), indicating a similar number of glutamatergic terminals in the PRC. Treatment with R13 restored the number of PSD-95-positive immunoreactive puncta in *Cdkl5* -/Y mice, but did not affect immunoreactivity for PSD-95 in *Cdkl5* +/Y mice (Figure 8A,B) or for VGlut1 in *Cdkl5* +/Y and *Cdkl5* -/Y mice (Figure 8A,C). Vehicle treatment did not affect immunoreactivity for PSD-95 or VGlut1 in *Cdkl5* +/Y and *Cdkl5* -/Y mice (Figure 8A,C).

The TrkB/PLCγ1 pathway is involved in diverse postsynaptic events, such as the modulation of AMPAR expression and trafficking, which in turn contributes to synaptic plasticity (Jang et al., 2013). As previously observed in hippocampal neurons (Tramarin et al., 2018), we found a lower expression of GluA2-AMPAR in the PRC of *Cdkl5* -/Y mice in comparison with wild-type mice (Figure 8D,E). Notably, GluA2-AMPAR levels in *Cdkl5* -/Y mice were rescued by treatment with R13 (Figure 8D,E).

### Altered Visual Recognition Memory in *Cdkl5* KO Mice Is Rescued by Treatment With the TrkB Agonist R13

**FIGURE 9:**
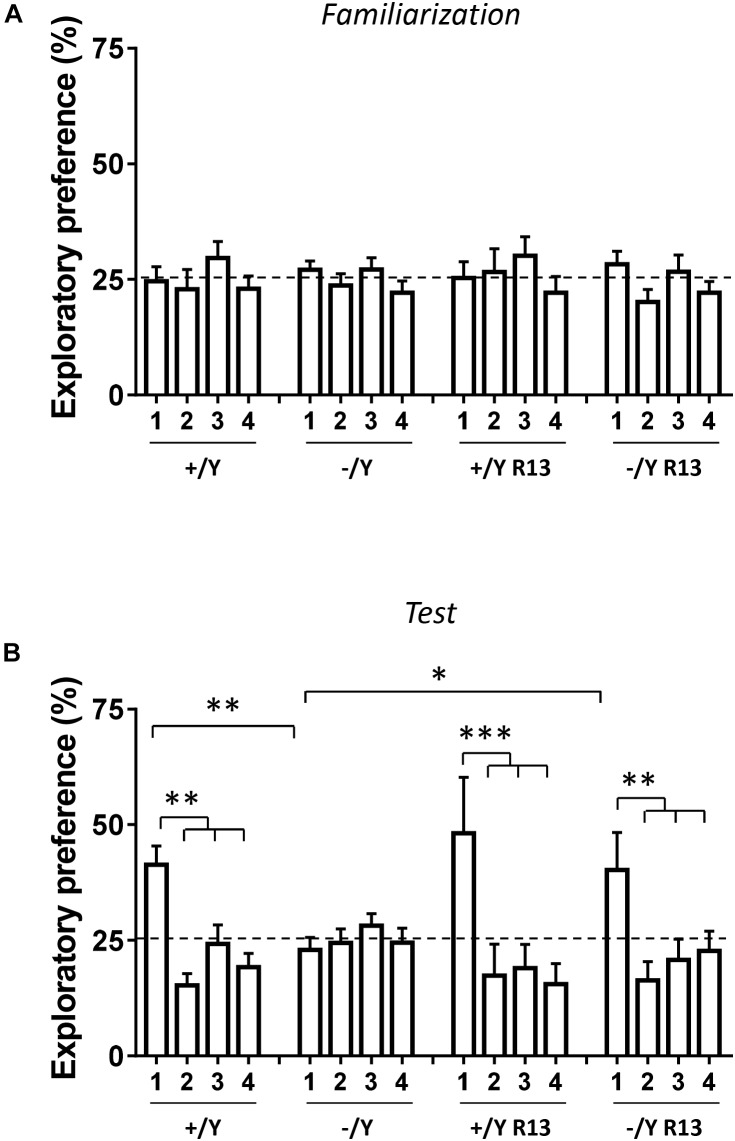
Effect of treatment with R13 on novel object recognition memory in *Cdkl5* -/Y mice. **(A)** Familiarization phase. Percentage of time spent exploring the four identical objects during the familiarization phase (10 min duration) of vehicle-treated *Cdkl5* -/Y (*n* = 13) and *Cdkl5* +/Y (*n* = 13) mice, and R13-treated *Cdkl5* -/Y (*n* = 9) and *Cdkl5* +/Y (*n* = 9) mice. **(B)** Exploratory Preference Test phase (one hour after the familiarization phase, 10 min duration). Percentage of time spent exploring new object 1 (that replaced the previous object 1) and the previous identical objects 2–4. Same mice as in **(A)**. Values represent mean ± SE. ^∗^*p* < 0.05; ^∗∗^*p* < 0.01; ^∗∗∗^*p* < 0.001 (Fisher’s LSD test after two-way ANOVA).

In the rodent brain, PRC plays an essential role in visual object recognition memory, that can be evaluated using the NOR test. A 4-object NOR test was performed in an open-field arena, preceded by a 2-day habituation phase (Supplementary Figure 1A). During the familiarization phase, animals normally show equal preference for the objects that are placed in an arena, while they exhibit a higher preference for the new object during the subsequent test phase. Discrimination among the objects (Preference Index) was calculated by taking into account the percentage of time spent exploring any of the four objects over the total time spent exploring the objects. As expected, the preference index in wild-type (+/Y) mice was significantly larger for the novel object during the test phase (Figure 9A,B). However, we did not observe any increase in preference index in *Cdkl5* -/Y mice (Figure 9B), indicating a deficit in remembering the identity of an object in an open field. Interestingly, treatment with R13 rescued visual recognition memory in *Cdkl5* -/Y mice (Figure 9B). R13 treatment did not affect locomotor activity in *Cdkl5* -/Y or *Cdkl5* +/Y mice (Supplementary Figure 1B); nor was it able to further increase the preference index in *Cdkl5*+/Y mice (Figure 9B).

## Discussion

### Functional and Morphological Impairments in the Perirhinal Cortex of *Cdkl5* KO Mice

Our results indicate that the magnitude of LTP is reduced in the PRC of *Cdkl5* KO mice, consistently with the strong reduction previously observed in the somatosensory cortex (Della Sala et al., 2016). To investigate the molecular mechanisms underlying LTP reduction, we analyzed some elements of the signaling cascade activated by BDNF. Previous studies in the hippocampus have shown that the PLCγ1 pathway mediated by TrkB plays a predominant role in LTP (Minichiello et al., 2002), whereas the Erk/Akt pathway is of lesser, if of any, importance (Korte et al., 2000). Accordingly, we found a lower PLCγ1 phosphorylation in the PRC of *Cdkl5* KO mice in comparison with wild-type mice, and no significant differences in Erk and Akt phosphorylation. This evidence suggests that the TrkB/PLCγ1 pathway plays a critical role in the observed alteration of LTP in the PRC.

Brain-derived neurotrophic factor levels were not reduced in *Cdkl5* KO mice, suggesting a specific effect of *Cdkl5* deletion on the autophosphorylation process at position Tyr816, rather than on BDNF availability.

At variance with the results obtained in the PRC (present data) and somatosensory cortex (Della Sala et al., 2016), LTP was previously found to be slightly increased in the hippocampal CA1 region of *Cdkl5* KO mice (Okuda et al., 2017), suggesting that CDKL5 action might be region-specific. These conflicting results might also be explained, at least in part, by different sensitivity to LTP inducing protocols, TBS [present data, and (Della Sala et al., 2016)] versus HFS (Okuda et al., 2017).

Previous studies in *Cdkl5* KO mice have shown reduced neuronal branching accompanied by reduced spine density and maturation in the hippocampus, in the visual cortex, and in the somatosensory cortex (Amendola et al., 2014; Fuchs et al., 2014; Della Sala et al., 2016; Pizzo et al., 2016; Trazzi et al., 2016). Accordingly, we found that PRC neurons of *Cdkl5* KO mice are characterized by a shorter dendritic length, a reduced number of branches, a lower spine density and a higher percentage of immature spines. Moreover, we observed a reduced number of PSD-95 immunoreactive puncta in dendritic spines. Since PSD-95 localization in the spine correlates with activity-driven synapse stabilization (Ehrlich et al., 2007), the decreased number of PSD-95 puncta in *Cdkl5* KO mice correlates with the observed increased number of immature spines. Evaluation of the number of presynaptic excitatory terminals (VGlut1 immunopositive puncta) showed no difference between *Cdkl5* KO and wild-type mice, in accordance with the observation that basic properties of synaptic function are unaltered in the PRC (present data) and in the hippocampus (Okuda et al., 2017). As observed in hippocampal neurons (Tramarin et al., 2018), we found a lower expression of GluA2-AMPAR in the PRC of *Cdkl5* -/Y mice. Most neuronal AMPARs contain this critical subunit, but in certain restricted neuronal populations and under certain physiological or pathological conditions, AMPARs that lack this subunit are expressed. This subunit determines many of the major biophysical properties of AMPARs (including Ca^2+^ permeability, single-channel conductance, and receptor kinetics), strongly influences receptor assembly and trafficking, and plays pivotal roles in various forms of synaptic plasticity (Isaac et al., 2007). In particular, GluA2-AMPAR is required for spine changes during synaptic plasticity (Asrar and Jia, 2013). Thus, it is reasonable to suppose that the lower expression of GluA2-AMPAR in the PRC might play a role in the impaired functional and morphological synaptic plasticity observed in the PRC of *Cdkl5* -/Y mice.

Visual recognition memory is the ability to judge the prior occurrence of stimuli and is fundamental to our ability to record events and to guide prospective behavior. Studies in humans and animals indicate that PRC plays an essential role in recognition memory and familiarity discrimination for individual items (Kealy and Commins, 2011; Suzuki and Naya, 2014; Brown and Banks, 2015). Our finding that short-term object recognition memory is impaired in *Cdkl5* KO mice is in line with the observed neuroanatomical defects and LTP impairment in the PRC. This behavioral evidence suggests that specific PRC defects may contribute, along with hippocampal impairments, to poor memory performance in individuals with CDD.

### Treatment With the TrkB Agonist R13 Rescues Functional and Morphological Impairments in the Perirhinal Cortex of *Cdkl5* KO Mice

The natural flavonoid 7,8-DHF, a potent small molecular TrkB agonist, displays beneficial effects on the brain in health and disease (Spencer, 2008; Williams and Spencer, 2012; Roncacé et al., 2017), but has only modest oral bioavailability and a moderate pharmacokinetic profile. To efficiently mimic the actions of BDNF, we used the recently synthesized 7,8-DHF prodrug R13, which is hydrolyzed into 7,8-DHF in liver microsomes and is characterized by a longer half-life and a higher plasma concentration, and higher brain exposure (Chen et al., 2018). We found that treatment with R13 activated TrkB/PLCγ1 signaling, rescued the impaired TBS-induced LTP, restored dendritic pattern as well as PSD-95 and GluA2-AMPAR levels, and improved the balance between immature and mature spines in PRC neurons of *Cdkl5* KO mice. Importantly, restoration of morphological and synaptic impairments leaded to recovery of PRC-dependent visual recognition memory in *Cdkl5* KO mice.

In wild type +/Y mice, treatment with R13 caused an activation of TrkB/PLCγ1 signaling in the PRC (Figure 4D), which, however, did not induce effects on PRC neuroanatomy or synaptic function and, consequently, on behavior. The finding that treatment with R13 has relatively scarce or no effects in normal animals is in line with a previous evidence (Garcia-Diaz Barriga et al., 2017) and suggests that TrkB/PLCγ1 signaling activation may help brain development under abnormal, but not normal, brain conditions.

Indeed, an increasing body of evidence suggests that PLCγ1 plays a pivotal role in the regulation of synaptic plasticity and maturation; in particular, PLCγ1 signaling is required for structural and functional changes in spine actin, PSD scaffolding, and AMPAR trafficking (Horne and Dell’Acqua, 2007). Consistent with its critical role, abnormal expression and activation of PLCγ1 has been observed in various brain disorders (Jang et al., 2013).

## Conclusion

In conclusion, present results provide the first evidence for morphological and functional impairments in the PRC of *Cdkl5* KO mice, associated with a deficit in visual recognition memory. The TrkB agonist R13 rescued most of these alterations, including LTP and visual recognition memory impairments, by triggering the TrkB/PLCγ1 pathway. If R13 induced a widespread positive effect in other brain areas, it might represent a promising candidate for a targeted therapeutic strategy aimed at restoring synaptic development and plasticity in CDD patients.

## Ethics Statement

Experiments were performed in accordance with the European Communities Council Directive of 24 November 1986 (86/609/EEC) for the use of experimental animals, and were approved by the Italian Ministry of Public Health (approval in 114/2018-PR). All efforts were made to minimize animal suffering and to keep the number of animals used to a minimum.

## Author Contributions

EC, GA, ST, and VR designed the study. ER, VR, ST, CF, GM, LG, ML, and GG performed the experiments. ER, VR, ST, and RR analyzed the data. KY executed the R13 synthesis. EC and GA wrote the manuscript.

## Conflict of Interest Statement

The authors declare that the research was conducted in the absence of any commercial or financial relationships that could be construed as a potential conflict of interest.
