# Supplementary material for: Functional and Structural Impairments in the Perirhinal Cortex of a Mouse Model of CDKL5 Deficiency Disorder Are Rescued by a TrkB Agonist
Source: Front Cell Neurosci. 2019 Apr 30;13:169. doi: 10.3389/fncel.2019.00169 (PMC6503158; doi:10.3389/fncel.2019.00169)
Supplement: Supplementary file 1 [file Table_1.DOCX]

| **ANTIBODIES** | **DILUTION** | **SOURCE** | **IDENTIFIER** |
| --- | --- | --- | --- |
| **Primary antibodies** | | | |
| Rabbit polyclonal anti-BDNF | 1:500 | Santa Cruz Biotechnology | Cat# sc 546 |
| Rabbit polyclonal anti-TrkB | 1:500 | Santa Cruz Biotechnology | Cat# sc 12 |
| Rabbit polyclonal anti-phospho-TrkB (Tyr 816) | 1:500 | Merck Millipore | Cat# abn1381 |
| Rabbit polyclonal anti-phospho-TrkB (Tyr 515) | 1:500 | Abcam | Cat# ab10968 |
| Rabbit polyclonal anti-phospho-PLCγ1 (Tyr 783) | 1:1000 | Cell Signaling | Cat# 2821 |
| Rabbit polyclonal anti-PLCγ1 | 1:1000 | Cell Signaling | Cat# 5690 |
| Rabbit polyclonal anti-AMPA receptor GluR2 | 1:1000 | Cell Signaling | Cat# 5306 |
| Rabbit polyclonal anti-phospho-Akt (Ser473) | 1:1000 | Cell Signaling | Cat# 4060 |
| Rabbit polyclonal anti-Akt | 1:1000 | Cell Signaling | Cat# 4691 |
| Rabbit polyclonal anti-phospho-Erk1/2 (Tyr202/Tyr204) | 1:1000 | Cell Signaling | Cat# 9101 |
| Mouse monoclonal anti-Erk1/2 | 1:1000 | Cell Signaling | Cat# 9107 |
| Rabbit polyclonal anti-GAPDH | 1:5000 | Sigma-Aldrich | Cat# G9545 |
| **Secondary antibodies** | | | |
| HRP-conjugated goat anti-mouse IgG | 1:5000 | Jackson Immuno Research | Cat# 115-035-003 |
| HRP-conjugated goat anti-rabbit IgG | 1:5000 | Jackson Immuno Research | Cat# 111-035-003 |

**Supplementary Table**
