# Supplementary material for: Functional and Structural Impairments in the Perirhinal Cortex of a Mouse Model of CDKL5 Deficiency Disorder Are Rescued by a TrkB Agonist
Source: Front Cell Neurosci. 2019 Apr 30;13:169. doi: 10.3389/fncel.2019.00169 (PMC6503158; doi:10.3389/fncel.2019.00169)
Supplement: Supplementary file 2 [file Data_Sheet_1.PDF]

A

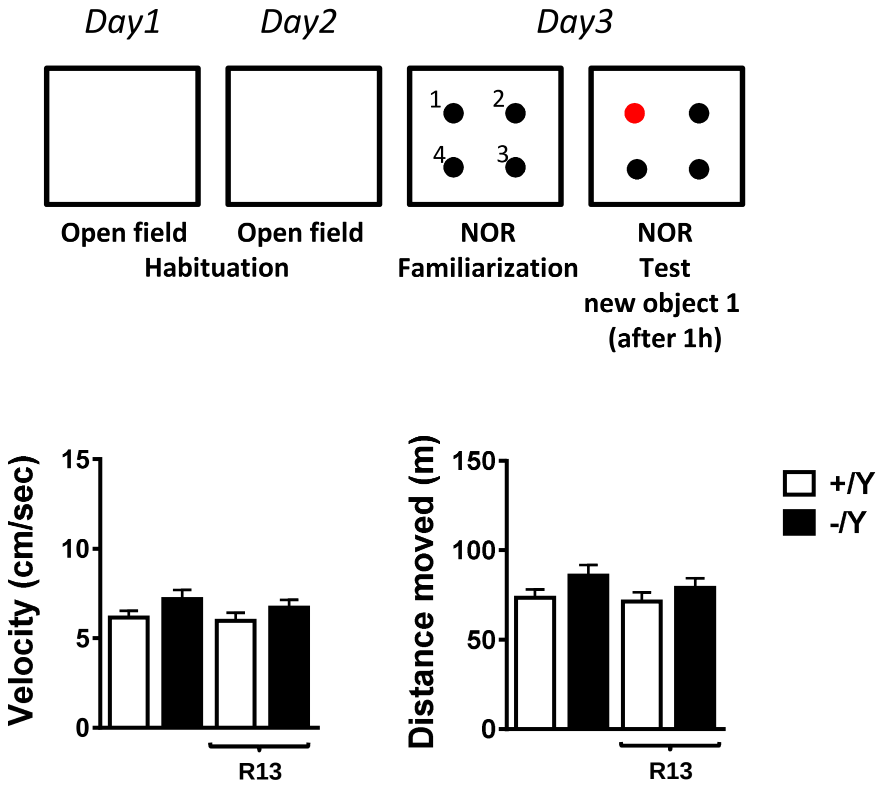

Supplementary Figure 1

A: Mice were exposed to an open field arena for 20 min on two consecutive days. On the third day, each mouse was exposed to four objects during a 10-min sample phase. After 1 h delay, during which one of the objects (object 1) was replaced with a novel object, the mouse was returned to the arena for the test phase. **B:** Locomotor activity measured as average locomotion velocity (left graph) and total distance traveled (right graph) during the second day 20-min open-field test in vehicle-treated Cdkl5 +/Y (n = 13) and Cdkl5 -/Y (n = 13) mice, and R13-treated Cdkl5 +/Y (n = 9) and Cdkl5 -/Y (n = 9) mice. Values represent mean ± SE. * $p < 0.05$; ** $p < 0.01$; *** $p < 0.001$ (Fisher's LSD test after two-way ANOVA).
