# Supplementary material for: Functional and Structural Impairments in the Perirhinal Cortex of a Mouse Model of CDKL5 Deficiency Disorder Are Rescued by a TrkB Agonist
Source: Front Cell Neurosci. 2019 Apr 30;13:169. doi: 10.3389/fncel.2019.00169 (PMC6503158; doi:10.3389/fncel.2019.00169)
Supplement: Supplementary file 3 [file Data_Sheet_2.pdf]

SUPPLEMENTARY DATA

MATERIALS AND METHODS

Electrophysiology

Slice preparation: Mice were deeply anesthetized with isoflurane before decapitation. The brain was quickly removed and immerged in ice-cold artificial cerebrospinal fluid (aCSF) composed of (in mM): NaCl, 124; KCl, 4.4; $NaHCO_3$, 26.2; NaH_2PO_4, 1; $CaCl_2$, 2.5; $MgCl_2$, 1.3; d-glucose, 10; L-ascorbic acid, 2; continuously gassed with 95% O_2 - 5% CO_2 and adjusted for pH 7.4. Horizontal brain slices (400 μm-thick), including the PRC, the entorhinal cortex, and the hippocampus were obtained from each hemisphere using a vibrating microtome. Once obtained, the slices were left to recover for at least 60 min at room temperature in aCSF saturated with 95% O_2 - 5% CO_2, and then stored in the same solution until usage (Aicardi et al., 2004).

Electrophysiological recordings: After the recovery period, a single slice was transferred to a submersion recording chamber and perfused at the rate of 3 ml/min with aCSF maintained at 32 ± 0.2°C and saturated with 95% O_2 - 5% CO_2. Recordings started 30 min after the slice was placed into the chamber. Extracellular recording electrodes consisted of borosilicate glass capillaries filled with aCSF (2–8 MΩ); stimulating electrodes were concentric bipolar electrodes (70–80 kΩ). The recording electrode was placed in layer II–III of the PRC, close to the rhinal sulcus and connected to a DC amplifier by an Ag/AgCl electrode. The stimulating electrode was inserted into the superficial layers (II–III) of the PRC at 400-500 μm from the recording electrode, in the rostral direction (temporal side of the rhinal fissure). Constant-current square pulses (0.2 ms, 20–220 μA, 0.033 Hz) were applied using a stimulus generator (Master 8, AMPI, Jerusalem, Israel) connected through a stimulus isolation unit to the concentric bipolar electrode. Field excitatory postsynaptic potentials (fEPSPs) evoked in layers II–III of the PRC by stimuli of increasing strength (20–220 μA) delivered at 0.033 Hz were recorded in order to obtain the input-output relations of the fEPSPs. Then, stimulus intensity was adjusted to induce 50% of the maximal synaptic response (Aicardi et al., 2004; Roncacé et al., 2017). To evaluate the effects of paired stimuli, a pair of stimuli with an interpulse interval of 200 ms was delivered. In LTP experiments, after at least 10 min of stable baseline recording, theta burst stimulation (TBS; four trains every 15 s, each train comprising 10 bursts of 5 pulses at 100 Hz, inter-burst interval 150 ms) (Ziakopoulos et al., 1999) was used to induce LTP. Responses were recorded at 0.5 min intervals for 10 min before, and for at least 60 min

after, LTP induction. LTP was defined as an increase in fEPSP amplitude of at least 10% at 25–30 min after TBS, and for the remainder of the recording. Synaptic plasticity was further investigated by delivering four consecutive TBS stimulations at 15-min intervals (Weng et al., 2011).
Measurements: The field excitatory postsynaptic potentials (fEPSPs) evoked in the PRC consisted of a negative going field potential, representing the excitatory synaptic response evoked by fibers distributed in the superficial layers. This potential was preceded by a negative-positive-negative fast wave, representing the compound action potential of the stimulated fibers (presynaptic volley; Fig. 1A). For reconstruction of the input-output relations, the amplitude of the presynaptic volley was measured from its initial positive peak to its negative peak, and the amplitude of the synaptic response was measured from the baseline to the maximum negativity (Fig. 1A). The paired-pulse ratio (PPR) was calculated as the peak amplitude of the second synaptic response (R2) divided by the peak amplitude of the first response (R1). For the evaluation of LTP, the peak amplitude of the field potential was measured, and any change after TBS was expressed in relation to the normalized pre-conditioning baseline (mean of the fEPSP amplitudes recorded in the last 5 min before TBS).

Golgi staining

Measurement of the dendritic tree: Golgi-stained PRC neurons located in layers II–III (10-15 per animal) were traced using dedicated software that was custom-designed for dendritic reconstruction (Immagini Computer, Milan, Italy), interfaced with Image Pro Plus. The dendritic tree was traced live, at a final magnification of 500x, by focusing into the depth of the section. Using this method the operator starts with branches emerging from the cell soma and, after having drawn the first parent branch (order 1, i.e., primary dendrites), goes on with all daughter branches of the next order in a centrifugal direction (Guidi et al., 2013). At the end of tracing, the program reconstructs the number and length of individual branches of each order, the mean length of branches of each order, and total number of branches and total dendritic length.
*Spine density and morphology***:** In Golgi-stained sections, spines of apical and basal dendrites of neurons of the PRC located in layers II–III (Fig. 5A,6A) were counted using a 100× oil immersion objective lens (1.4 NA). In each mouse, fifteen segments were analyzed. The linear spine density was calculated by dividing the total number of counted spines by the length of the sampled dendritic segment. Spine density was expressed as the number of spines per 10 μm dendrite. Dendritic spines can have a different shape morphology based on their maturation. The number of spines belonging to the 2 different groups (immature spines: filopodium-like, thin- and stubby-shaped; mature spines: mushroom- and cup-shaped) was counted and expressed as a percentage (Risher et al., 2014). About 200–250 spines from 25–30 dendrites derived from 10–20 neurons were analyzed per condition.

REFERENCES

Aicardi, G., Argilli, E., Cappello, S., Santi, S., Riccio, M., Thoenen, H., et al. (2004). Induction of long-term potentiation and depression is reflected by corresponding changes in secretion of endogenous brain-derived neurotrophic factor. Proc Natl Acad Sci U S A 101(44), 15788-15792. doi: 10.1073/pnas.0406960101

Guidi, S., Stagni, F., Bianchi, P., Ciani, E., Ragazzi, E., Trazzi, S., et al. (2013). Early pharmacotherapy with fluoxetine rescues dendritic pathology in the Ts65Dn mouse model of down syndrome. Brain Pathol 23(2), 129-143. doi: 10.1111/j.1750-3639.2012.00624.x

Risher, W.C., Ustunkaya, T., Singh Alvarado, J., and Eroglu, C. (2014). Rapid Golgi analysis method for efficient and unbiased classification of dendritic spines. PLoS One 9(9), e107591. doi: 10.1371/journal.pone.0107591

Roncacé, V., Burattini, C., Stagni, F., Guidi, S., Giacomini, A., Emili, M., et al. (2017). Neuroanatomical alterations and synaptic plasticity impairment in the perirhinal cortex of the Ts65Dn mouse model of Down syndrome. Neurobiol Dis 106, 89-100. doi: 10.1016/j.nbd.2017.06.017

Weng, S.M., McLeod, F., Bailey, M.E., and Cobb, S.R. (2011). Synaptic plasticity deficits in an experimental model of rett syndrome: long-term potentiation saturation and its pharmacological reversal. Neuroscience 180, 314-321. doi: 10.1016/j.neuroscience.2011.01.061

Ziakopoulos, Z., Tillett, C.W., Brown, M.W., and Bashir, Z.I. (1999). Input-and layer-dependent synaptic plasticity in the rat perirhinal cortex in vitro. Neuroscience 92(2), 459-472. doi: 10.1016/s0306-4522(98)00764-7
